# *De novo* assembly of bacterial transcriptomes from RNA-seq data

**DOI:** 10.1186/s13059-014-0572-2

**Published:** 2015-01-13

**Authors:** Brian Tjaden

**Affiliations:** Computer Science Department, Wellesley College, Wellesley, MA 02481 USA

## Abstract

Transcriptome assays are increasingly being performed by high-throughput RNA sequencing (RNA-seq). For organisms whose genomes have not been sequenced and annotated, transcriptomes must be assembled *de novo* from the RNA-seq data. Here, we present novel algorithms, specific to bacterial gene structures and transcriptomes, for analysis of bacterial RNA-seq data and *de novo* transcriptome assembly. The algorithms are implemented in an open source software system called Rockhopper 2. We find that Rockhopper 2 outperforms other *de novo* transcriptome assemblers and offers accurate and efficient analysis of bacterial RNA-seq data. Rockhopper 2 is available at http://cs.wellesley.edu/~btjaden/Rockhopper.

**Electronic supplementary material:**

The online version of this article (doi:10.1186/s13059-014-0572-2) contains supplementary material, which is available to authorized users.

## Introduction

High-throughput RNA sequencing (RNA-seq) is being used increasingly for transcriptome assays [1]. One of the challenges for studies employing RNA-seq experiments is efficient and reliable extraction of transcriptomic insights from the wealth of RNA-seq data. Often, following RNA-seq experiments, the large resulting data sets are subjected to various stages of computational analysis, such as quality control, normalization, transcriptome assembly, quantification of transcript abundance, and testing for differential gene expression under various conditions [2]. Analysis of the data can be a bottleneck in RNA-seq studies owing to the size of the data, the complexity of the analysis, and a lack of user-friendly software tools.

In particular, assembling transcripts is often a core stage of RNA-seq data analysis, yet efficient and accurate transcriptome assembly remains a challenging problem owing to a variety of factors, including artifacts from library construction, errors in sequencing, variable intra-read and inter-read error rates, repeat sequences, and transcript expression ranges that span several orders of magnitude [3]. Most approaches for assembling transcripts from short read sequences relate to one of two families: reference-based assembly and *de novo* assembly [4]. Reference-based assembly involves aligning sequencing reads to a sequenced reference genome. Reference-based assembly is generally preferable when a high-quality genome sequence is available since reference-based approaches are fast and relatively precise. *De novo* assembly involves assembling transcripts from sequencing reads by combining overlapping reads. *De novo* assembly is necessary when a high-quality reference genome is unavailable, such as for many non-model organisms, when analyzing complex microbial communities, in metatranscriptome studies, and when investigating unculturable microorganisms.

A number of mature computational tools exist for both reference-based transcriptome assembly [5-7] and *de novo* transcriptome assembly [8-11]. However, most of the aforementioned tools were designed primarily for eukaryotic transcriptomes. Bacterial transcriptome assembly faces different challenges than eukaryotic transcriptome assembly. For example, bacterial genomes are generally denser than eukaryotic genomes and neighboring bacterial transcripts frequently overlap, making it challenging to distinguish the boundaries of neighboring bacterial transcripts. Polycistronic messages further complicate bacterial transcriptome assembly, particularly when different promoters of an operon are employed under different conditions. Also, models for noncoding RNAs in eukaryotes are generally inappropriate for the small regulatory RNAs common in bacteria.

**Figure 1:**
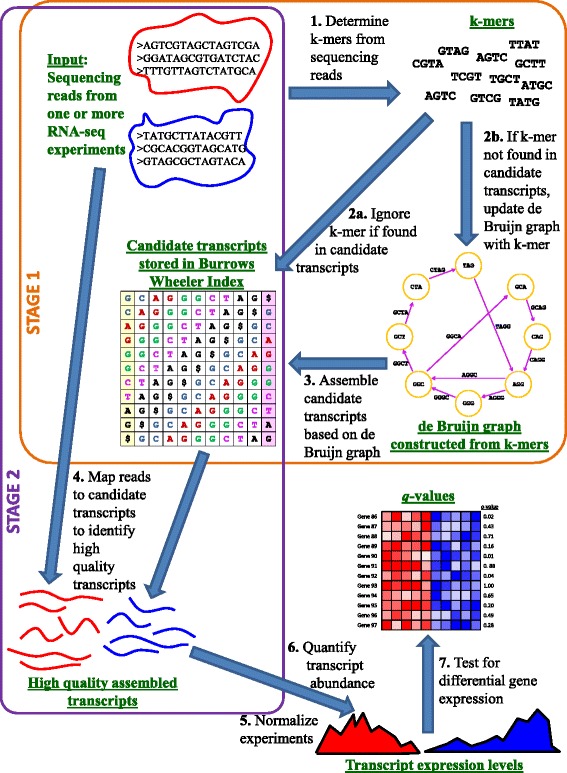
**Rockhopper 2 workflow depicting the various phases of Rockhopper 2’s analyses.** As input, Rockhopper 2 requires one or more files of sequencing reads from RNA-seq experiments. In the first analysis stage, Rockhopper 2 determines *k*-mers from the sequencing reads and builds a de Bruijn graph from the *k*-mers. The de Bruijn graph is used to assemble candidate transcripts, which are stored in a Burrows-Wheeler index. In the second analysis stage, Rockhopper 2 aligns the sequencing reads to the assembled candidate transcripts to determine a final set of high-quality assembled transcripts. After the second stage, transcriptome assembly is complete and Rockhopper 2 performs several downstream analyses, including normalizing data from different experiments, quantifying transcript abundance, and testing for differential gene expression across multiple conditions.

In an attempt to address the paucity of computational methods for assembling bacterial transcriptomes from RNA-seq data, we previously developed Rockhopper [12], a system that supports reference-based assembly of bacterial transcriptomes. In the current study, we have developed novel algorithms for *de novo* assembly of bacterial transcriptomes, which we have implemented in the system Rockhopper 2. We show that our algorithms for *de novo* assembly of bacterial transcriptomes outperform other leading approaches, in terms of both sensitivity and specificity. Further, our algorithms offer dramatic improvements in efficiency, so that our *de novo* assembly is comparable to reference-based assembly in terms of execution time. While many *de novo* assemblers require high-performance computing platforms, Rockhopper 2 has been designed with limited resource requirements so that it performs effectively on common laptop machines. In addition to *de novo* transcriptome assembly, Rockhopper 2 is a comprehensive system that supports the various stages of RNA-seq data analysis, including normalizing data from different experiments, quantifying transcript abundance, and testing for differential transcript expression. Details of the Rockhopper 2 workflow are illustrated in Figure 1. Finally, we developed Rockhopper 2 with user-friendliness in mind, so that it would be accessible to a broad range of scientists that use bacterial RNA-seq experiments in their investigations. Rockhopper 2 is open-source software implemented in Java, released under the GNU GPL license, and is available for all major platforms at [13,14].

## Materials and methods

### Assembly algorithm

As input, Rockhopper 2 requires one or more files of sequencing reads. Sequencing read files may be in fastq, qseq, fasta, sam, or bam format [15]. Files in fastq, qseq, or fasta format optionally may be gzipped. Rockhopper 2 works with single-end reads as well as paired-end reads, and reads may be strand-specific or strand-ambiguous.

*De novo* transcriptome assembly in Rockhopper 2 proceeds in two stages (Figure 1). In the first stage, candidate transcripts are assembled from *k*-mers found in the sequencing reads (*k* = 25 by default). After the first stage, every *k*-mer in an assembled candidate transcript will correspond to at least one *k*-mer from a sequencing read. However, candidate transcripts may not be supported by full-length reads. Thus, in a second stage, sequencing reads are mapped to candidate transcripts in order to filter candidate transcripts into a set of high quality final transcripts that are well supported by full-length sequencing reads. Algorithmic details of each stage are provided below.

In the first stage of *de novo* transcriptome assembly, Rockhopper 2 maintains two data structures, a de Bruijn graph [16,17] and a Burrows-Wheeler index [18,19]. While de Bruijn graphs are common among *de novo* assemblers [4,17], Burrows-Wheeler indices are not. Instead, Burrows-Wheeler indices are common in reference-based assemblers [2]. But it is precisely the integration of the two structures, working in concert, that enables Rockhopper 2’s speed and minimal memory usage, distinguishing it from other *de novo* assemblers. Both data structures are initially empty and are populated as sequencing reads are processed in the first stage. The de Bruijn graph is implemented with a hash table, where *k*-mer graph edges are stored as keys in the table and *k*-mer edge occurrences are stored as values in the table. Graph nodes are stored implicitly. The Burrows-Wheeler index keeps track of assembled candidate transcripts. For each sequencing read, its set of *k*-mers is determined. If a *k*-mer already occurs in the Burrows-Wheeler index, that is, is already part of an assembled candidate transcript, then the *k*-mer is not considered further. If the *k*-mer is not part of an assembled candidate transcript, then the de Bruijn graph is updated with the *k*-mer.

As *k*-mers are added to the de Bruijn graph, it grows in size. As more memory is consumed and the amount of available memory approaches zero, Rockhopper 2 reduces the size of the de Bruijn graph by assembling candidate transcripts. Paths through the graph are traversed, beginning with the most frequently occurring edges. For each edge with frequency at least α, a path is started and greedily extended if a neighboring edge can be found with frequency at least β (default values α = 50 and β = 5 were determined empirically). When an edge is traversed, it is removed from the graph. A path corresponds to an assembled candidate transcript. When a path is extended as far as possible, the corresponding assembled candidate transcript is added to the Burrows-Wheeler index.

A de Bruijn graph has 4^*k*^ potential edges, which requires more memory to store than is available on most personal computers. As RNA-seq experiments continue to generate increasing amounts of sequencing data, this limit will be approached in de Bruijn graph-based assemblers, unless sequencing error rates drop dramatically. Thus, most assemblers require high-performance computing hardware with enhanced memory resources. Rockhopper 2 takes a different approach and limits the size of de Bruijn graphs. Rockhopper 2’s approach has two main advantages: it enables the system to run on common personal computers and it quickly channels resources away from low frequency *k*-mers that are likely to correspond to sequencing errors or other artifacts.

In the first stage, we assemble candidate transcripts and store them in a Burrows-Wheeler index. In the second phase, we make a second pass through the sequencing reads, aligning each read to the index. Importantly, the Burrows-Wheeler index allows for rapid alignment in that a read of length *m* can be aligned to the set of candidate transcripts with total length *N* in *O*(*m*) time with small constants. In the case of paired-end reads, we require that the paired-ends for each read form a scaffold consistent with the transcript. We keep track of how many full-length reads align to each candidate transcript and at what loci. Sufficiently long regions of candidate transcripts are retained as high quality finalized transcripts if at least ε reads align throughout the length of the region (ε = 20 by default).

Following *de novo* assembly of high quality transcripts, Rockhopper 2 proceeds with several post-assembly phases of analysis. To enable comparison between different samples and experiments, Rockhopper 2 normalizes each RNA-seq data set using upper quartile normalization [20]. Transcript abundance levels are estimated using a measure similar to RPKM (reads per kilobase per million), which sums the number of reads for a transcript and divides by the transcript’s length and a normalization factor [7]. While the total number of reads in the sample is often used to determine the RPKM normalization factor, Rockhopper 2 uses the more robust normalizer of upper quartile transcript expression [20]. Finally, Rockhopper 2 tests for differential transcript expression in pairs of conditions using the algorithm of DESeq [21]. In summary, Rockhopper 2 estimates the variance of a transcript’s expression, uses local regression to obtain a smooth estimate of the variance, and then performs a statistical test to determine whether a transcript shows differential expression in data from two or more conditions. The negative binomial distribution is used as the statistical model in order to compute a *P*-value indicating the probability of observing the transcript’s expression levels in the different conditions by chance. To correct for multiple tests across the set of transcripts, *P*-values are corrected and *q*-values are reported that control the false discovery rate using the Benjamini-Hochberg procedure [22].

### High-throughput sequencing data

*Escherichia coli* strain MG1655 was used in three biological replicate DNA-seq experiments (Cari Vanderpool, personal communication). Library construction and sequencing on an Illumina HiSeq 2500 were performed at the WM Keck Center for Comparative and Functional Genomics at the University of Illinois at Urbana-Champaign. The DNA libraries were prepared with the KAPA Library Preparation Kits (KAPA Biosystems (Wilmington, MA, USA)). The libraries were quantified by quantitative PCR , pooled in equimolar concentration, and sequenced on one lane for 101 cycles from one end of the fragments using a TruSeq SBS version 3 sequencing kit (Illumina (San Diego, CA, USA)). The fastq files were generated with Casava 1.8.2 (Illumina).

RNA-seq data from *E. coli*, *Streptococcus pyogenes*, *Mycobacterium tuberculosis*, *Bacillus subtilis*, *Staphylococcus aureus*, *Pyrococcus abyssi*, *Acinetobacter oleivorans*, *Propionibacterium acnes*, *Methanobrevibacter smithii*, *Clostridium acetobutylicum*, and *Deinococcus gobiensis* were downloaded from the Sequence Read Archive (SRA) [23]. Details on each RNA-seq data set, including accession number in the SRA, length of the reads, whether the reads are single-end or paired-end, and the number of reads, is provided in Table 1. The *Schizosaccharomyces pombe* RNA-seq data [24] were downloaded from the Trinity tutorial [25].

**Table 1:** **Sequencing data sets**

**Organism**	**Type**	**Domain**	**Class**	**SRA accession number**	**Read type**	**Length of reads (bp)**	**Number of reads**	**Number of reference genes**
*Escherichia coli*	DNA-seq	Bacteria	Gammaproteobacteria	SRP049375	Single	100	67,713,365	-
*Escherichia coli*	RNA-seq	Bacteria	Gammaproteobacteria	SRX254784	Single	100	34,085,732	4,190
*Acinetobacter oleivorans*	RNA-seq	Bacteria	Gammaproteobacteria	SRX560107	Paired	101	19,140,537	2,934
*Deinococcus gobiensis*	RNA-seq	Bacteria	Deinococci	SRX061110	Paired	75	18,676,333	610
*Mycobacterium tuberculosis*	RNA-seq	Bacteria	Actinobacteria	SRX380298	Paired	51	2,364,009	752
*Streptococcus pyogenes*	RNA-seq	Bacteria	Bacilli	SRX252449	Single	72	7,049,947	372
*Bacillus subtilis*	RNA-seq	Bacteria	Bacilli	SRX533166	Single	51	14,010,827	1,917
*Staphylococcus aureus*	RNA-seq	Bacteria	Bacilli	SRX172891	Paired	101	9,067,797	1,720
*Propionibacterium acnes*	RNA-seq	Bacteria	Actinobacteria	SRX278003	Single	75	195,541,304	1,777
*Clostridium acetobutylicum*	RNA-seq	Bacteria	Clostridia	SRX316281	Single	50	13,256,052	202
*Pyrococcus abyssi*	RNA-seq	Archaea	Thermococci	SRX556571	Single	40	51,342,770	133
*Methanobrevibacter smithii*	RNA-seq	Archaea	Methanobacteria	SRX031877	Single	36	32,744,832	211
*Schizosaccharomyces pombe*	RNA-seq	Eukarya	Schizosaccharomycetes	NA	Paired	68	4,000,000	3,591

The table summarizes the DNA-seq data set and the 12 RNA-seq data sets used in this study. Information in the table includes the length and number of sequencing reads in each data set. NA, not available.

### Performance evaluation

In order to evaluate Rockhopper 2’s performance, we compared it with two leading *de novo* transcriptome assemblers: Trinity version trinityrnaseq_r20140413p1 [8,25] and SOAPdenovo2 version 2.04 [10,26]. Default parameters were used for Trinity (Trinity --seqType fq -JM 10G -CPU 8) and SOAPdenovo2 (SOAPdenovo-63mer all -p 8 -d 49). All three assemblers were executed on the same hardware with the number of processors set to 8. The three software systems were used to assemble transcriptomes using sequencing data from 12 microorganisms with sequenced and annotated genomes, though the genomes and their annotations were not used by any of the software systems during assembly. The genome sequences and annotations were used only to evaluate the *de novo* assembled transcriptomes.

A variety of measures was used to evaluate the performance of the different assemblers [4,27]. In some cases, the correspondence between assembled transcripts and annotated genes is assessed. Since not all genes are likely to be expressed in a given experiment, the *de novo* assembled transcripts are compared not against all annotated genes but against a subset of annotated genes, which we call reference genes. A reference gene is defined as a gene where every *k*-mer in the gene sequence (*k* = 25) occurs in at least one sequencing read. Reference genes can possibly be reconstructed by the *de novo* assemblers whereas non-reference genes cannot. The set of reference genes is analogous to the Oracle Set used to evaluate the Trinity system [8].

Specificity is a measure that represents the percentage of assembled transcripts that align to the genome. Specificity can be expressed as (1.0 - False positive rate), where a false positive is an assembled transcript that does not align to the genome. Specificity is calculated as:

$$ \frac{{\displaystyle {\sum}_{t\in T}}I\left(\frac{\left|{a}_G^t\right|}{\left|t\right|}\ge \delta \right)}{\left|T\right|} $$

where *T* is the set of assembled transcripts and *G* is a genome. ${a}_G^t$ is the alignment of the sequence of *t* to the sequence of *G*, and $\left|{a}_G^t\right|$ is the length of the alignment. *I* is an indicator function with parameter *δ* set to 1.0.

Sensitivity represents the percentage of sequence from reference genes covered by assembled transcripts. Sensitivity in this context is sometimes referred to as the coverage or completeness of an assembler. Sensitivity is given by:

$$ \frac{{\displaystyle {\sum}_{g\in R}}\left|{g}^T\right|}{{\displaystyle {\sum}_{g\in R}}\left|g\right|} $$

where *R* is the set of reference genes. Following alignment of assembled transcripts in *T* to the set of reference genes *R*, |*g*^*T*^| is the number of nucleotides in the sequence of reference gene *g* that are covered via alignment by one or more transcripts from *T*. In the special case of assessing the quality of assemblies from DNA-seq data rather than RNA-seq data, sensitivity represents the percentage of sequence from the entire genome, rather than from reference genes, covered by assembled transcripts:

$$ \left|{G}^T\right|/\left|G\right| $$

Contiguity represents the percentage of reference genes that are at least δ = 80% covered by a single longest assembled transcript [4]. Contiguity is defined as:

$$ \frac{{\displaystyle {\sum}_{g\in R}}I\left(\frac{\left|{g}^{T^{\hbox{'}}}\right|}{\left|g\right|}\ge \delta \right)}{\left|R\right|} $$

where $\left|{g}^{T^{\hbox{'}}}\right|$ is the number of nucleotides in the sequence of reference gene *g* that are covered via alignment by the transcript from *T* that has the longest alignment to *g. I* is an indicator function with parameter *δ* set to 0.8.

RMBT (reads mapping back to transcripts) represents the percentage of sequencing reads that align to an assembled transcript. RMBT is given by:

$$ \frac{{\displaystyle {\sum}_{s\in S}}I\left(\frac{\left|{a}_T^s\right|}{\left|s\right|}\ge \delta \right)}{\left|S\right|} $$

where *S* is the set of sequencing reads, ${a}_T^s$ is the alignment of the read *s* to the set of assembled transcripts *T*, and $\left|{a}_T^s\right|$ is the length of the alignment. *I* is an indicator function with parameter *δ* set to 1.0.

Accuracy represents the percentage of correctly assembled bases; that is, for those transcripts that align to the genome, the accuracy is the percentage of perfect matches in the alignments (as opposed to mismatches or gaps). Accuracy is calculated as:

$$ \frac{{\displaystyle {\sum}_{t\in T}} PM\left({a}_G^t\right)}{{\displaystyle {\sum}_{t\in T}}\left|{a}_G^t\right|} $$

where $PM\left({a}_G^t\right)$ is the number of perfect matches in the alignment of the sequence *t* to the sequence of *G*, and $\left|{a}_G^t\right|$ is the length of the alignment. Efficiency represents the execution time of an assembler, as measured in minutes. Resourcefulness represents the amount of memory (RAM) required during an assembly.

## Results

In order to assess Rockhopper 2’s ability to assemble transcriptomes *de novo*, we executed Rockhopper 2 on high-throughput sequencing data from a variety of organisms whose genomes have been sequenced and annotated. The genome sequences and annotations were not used by Rockhopper 2, rather they allow us to evaluate Rockhopper 2 by investigating the correspondence between the *de novo* assembled transcriptomes it produces and the genome sequences and annotations. To provide points of comparison, two leading *de novo* transcriptome assemblers, Trinity [8,25] and SOAPdenovo2 [10,26], were executed on the same data, and their results are compared with those of Rockhopper 2.

### Genomic DNA-seq data

We used three biological replicates of genomic DNA-seq data from *E. coli* (see Materials and methods) for preliminary assessment of Rockhopper 2’s performance. Genome assembly based on DNA-seq data is generally more straightforward than transcriptome assembly based on RNA-seq data since RNA-seq data correspond to transcripts with highly variable expression levels and lengths, whereas DNA-seq data do not. Thus, the performance of an assembler using DNA-seq data can suggest an upper bound on the quality of the assembly that we can expect from the assembler using RNA-seq data.

**Figure 2:**
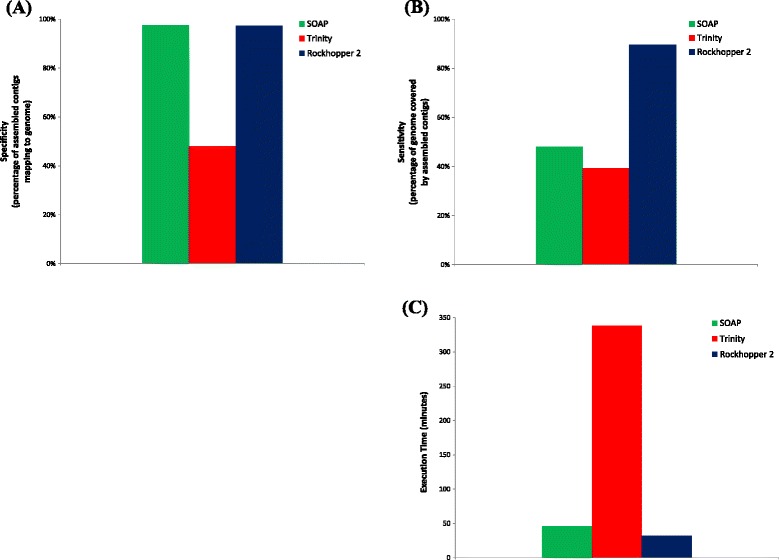
**Performance assembling** ***E. coli*** **genome from DNA-seq data.** The performance of Rockhopper 2 as well as two other assemblers, SOAPdenovo2 and Trinity, on three biological replicate DNA-seq experiments from *E. coli* is illustrated. **(A)** Specificity is the percentage of assembled contigs that align to the *E. coli* genome. **(B)** Sensitivity is the percentage of the *E. coli* genome sequence that is covered by assembled contigs aligning to the genome. **(C)** Execution time is the number of minutes that an assembler requires to execute on the DNA-seq data.

Figure 2 and Additional file 1 provide statistics on assemblies based on the DNA-seq data. Rockhopper 2 and SOAPdenovo2 both had close to 100% specificity, indicating that the vast majority of their assembled contigs could be aligned to the *E. coli* genome (Figure 2A). In contrast, just over half of the contigs assembled by Trinity were considered false positives in that they did not align to the *E. coli* genome (Figure 2A). Further, Rockhopper 2’s assembly had a sensitivity of approximately 90%, indicating that 90% of the genome was covered by Rockhopper 2’s assembled contigs (Figure 2B). While Rockhopper 2 was not designed as a genome assembler, these results suggest that it does a plausible job of reconstructing most of the *E. coli* genome. For comparison, the contigs assembled by SOAPdenovo2 and Trinity covered just under half the *E. coli* genome (Figure 2B). Regions of the genome that were not covered by contigs from any of the assemblers generally correspond to some combination of repeat regions, errors in sequencing reads, and biases during library construction and high-throughput sequencing. Finally, we found that Rockhopper 2 required about 32 minutes to generate its assembly, a rate comparable to that of SOAPdenovo2 and substantially faster than that of Trinity (Figure 2C).

### RNA-seq data

While DNA-seq data provide a starting point for understanding the quality of assemblies, the performance of an assembler using RNA-seq data is more meaningful. Thus, we gathered data from RNA-seq experiments conducted by 12 different labs for 12 different microorganisms (see Materials and methods). The organisms included nine bacteria, two archaea, and one fungus. While Rockhopper 2 was not designed for eukaryotic transcriptome assembly, we evaluated its performance on data from the fungus *S. pombe* primarily because this same data set was used by the authors of the Trinity assembler to assess Trinity’s performance [8,25]. The 12 organisms represented in our analysis were chosen to reflect a wide range of phylogenetic diversity in order to help us understand the robustness of our assemblies. The 12 RNA-seq data sets range in size from approximately 2 million reads to 200 million reads and include 7 sets of single-end sequencing reads and 5 sets of paired-end sequencing reads (Table 1).

**Figure 3:**
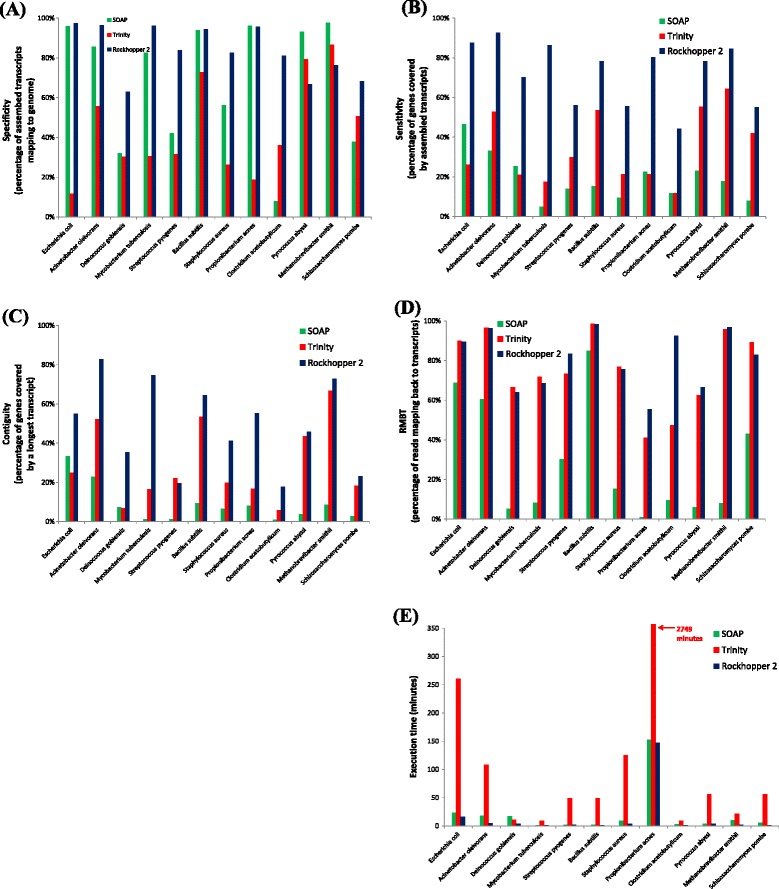
**Performance assembling transcripts from RNA-seq data.** The performance of each of three assemblers on 12 RNA-seq data sets is illustrated. The 12 RNA-seq data sets correspond to nine bacteria, two archaea, and one fungus. **(A)** Specificity is the percentage of assembled transcripts that align to the genome. **(B)** Sensitivity is the percentage of the reference gene sequences that is covered by assembled transcripts aligning to the reference genes. **(C)** Contiguity is the percentage of reference genes that are at least δ = 80% covered by their single longest aligning transcript. **(D)** RMBT is the percentage of sequencing reads to align to assembled transcripts. **(E)** Execution time is the number of minutes that an assembler requires to execute on the RNA-seq data set.

A variety of statistics (see Materials and methods) was used to evaluate the assemblies produced by Rockhopper 2, Trinity, and SOAPdenovo2 across the 12 RNA-seq data sets and the results are provided in Figure 3 and Additional file 1. Rockhopper 2 and SOAPdenovo2 generally had the highest specificity, generating fewer false positive assembled transcripts that did not align to the corresponding genome (Figure 3A). Interestingly, Rockhopper 2’s specificity was lowest among the three assemblers on the two archaea data sets, but otherwise was among the highest. Assembly of additional RNA-seq data sets from prokaryotes beyond the 11 used in this study will help illuminate whether Rockhopper 2’s higher specificity on bacterial data and lower specificity on archaeal data, relative to the other two assemblers, is a broad trend resulting from biases toward certain domains or if it is an artifact of a small sample size. In terms of sensitivity, Rockhopper 2 demonstrated the highest sensitivity of the three assemblers across the 12 data sets, with its assembled transcripts covering a significantly larger percentage of reference genes than those of the other two assemblers (Figure 3B). Contiguity reflects the percentage of reference genes covered by a single longest transcript and is a useful measure for distinguishing whether an assembly contains transcripts covering a gene with multiple short transcripts or a single long transcript. Rockhopper 2’s assemblies demonstrate greater contiguity than those of the other two assemblers for 11 of the 12 data sets, with Trinity’s assembly demonstrating the greatest contiguity for the *S. pyogenes* data set (Figure 3C). RMBT indicates the percentage of sequencing reads that align to assembled transcripts; this measure is often used to evaluate assemblers under the assumption that higher RMBT corresponds to a greater percentage of reads used in constructing an assembly, which is desirable in that it is more likely to lead to a robust assembly than using a smaller percentage of reads when generating an assembly. Trinity and Rockhopper 2 consistently had high RMBT, in contrast to SOAPdenovo2, suggesting that these two assemblers generally use the majority of sequencing reads to construct their assemblies (Figure 3D). Finally, the execution time of the assemblers was assessed. Both SOAPdenovo2 and Rockhopper 2 demonstrated substantially greater efficiency than Trinity across the 12 data sets (Figure 3E).

**Figure 4:**
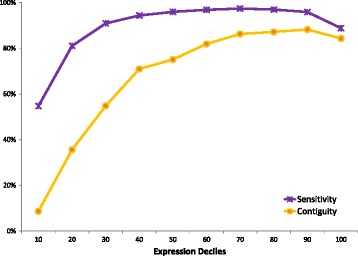
**Rockhopper 2 performance at different expression deciles.** For each of the 12 RNA-seq data sets, the set of reference genes was divided into 10 groups based on their expression levels, with the 10% of reference genes with lowest expression in the first group and the 10% of reference genes with highest expression in the last group. The sensitivity (purple) and contiguity (yellow) of Rockhopper 2’s assemblies across all 12 RNA-seq data sets are illustrated.

In order to investigate how Rockhopper 2’s assemblies are affected by expression level, we evaluated Rockhopper 2’s sensitivity and contiguity across the 12 RNA-seq data sets at different expression deciles (Figure 4). Each point represents an average across the 12 data sets (Figure 4). For example, the leftmost point corresponds to the average sensitivity (purple) or contiguity (yellow) of Rockhopper 2’s assemblies across the 12 data sets for the 10% least highly expressed reference genes. The rightmost point corresponds to the average sensitivity (purple) or contiguity (yellow) of Rockhopper 2’s assemblies across the 12 data sets for the 10% most highly expressed reference genes. For this analysis, rather than requiring assembled transcripts to align exactly to reference genes, we used a more permissive alignment criterion and allowed assembled transcripts to align to reference genes with a small number of gaps or mismatches (BLAST E-value <0.01). Unsurprisingly, Rockhopper 2 is better able, in terms of sensitivity and contiguity, to assemble transcripts with higher expression than lower expression, as performance generally improves as the expression decile increases (Figure 4). However, there is a small decrease in performance at the very highest expression deciles (Figure 4). This asymmetric rainbow-shaped curve is consistent with what others have observed [27], namely that assembly performance generally improves rapidly from the lowest expression quantiles to mid-level expression quantiles, plateaus across mid-level expression quantiles to higher-level expression quantiles, and decreases slightly at the highest level expression quantiles. These results provide one indication as to how confident a user can be in Rockhopper 2’s assembled transcripts for transcripts expressed at different levels.

We also considered a couple of additional measures of assembly performance. Accuracy is the percentage of correctly assembled bases and is computed by aligning assembled transcripts to the corresponding genome and calculating, for those transcripts that align, the percentage of bases in the alignment that correspond to perfect matches as opposed to mismatches or gaps [4]. All three assemblers showed accuracies greater than 99% across all 12 RNA-seq data sets (Additional file 1). Resourcefulness reflects the amount of RAM consumed during an assembly. A comparison of memory usage by SOAPdenovo2 and Trinity has been performed by others [27] and we did not repeat the analysis here. With default parameters, Rockhopper 2 uses at most 1.2 GB (gigabytes) of memory. For the assemblies in this study, we allowed Rockhopper 2 to use up to 2.0 GB of memory, an amount generally available on any common laptop. In contrast, the authors of the Trinity assembler recommend approximately 1 GB of memory per million paired reads for Trinity [25]. The RNA-seq data sets used in this study contained between 2 million and 195 million reads, with an average of 36 million reads. Thus, Trinity’s memory consumption typically requires high-performance computing hardware whereas Rockhopper 2 has no such requirement. All three assemblers are fully parallelizable and their runtime performance scales inversely with the number of processors available for computation in the machine on which the assembler is executed.

## Conclusions

Transcriptome assembly is a common step in the analysis of RNA-seq data. When a sequenced genome is available, assembly approaches can leverage the reference genome by aligning sequencing reads to the genome. When a high-quality reference genome is not available, transcriptomes must be assembled *de novo*. While a number of mature tools exist for *de novo* assembly of transcriptomes from RNA-seq data, these tools were designed primarily for eukaryotic data and their performance suffers when applied to bacterial data. In this study, we propose novel algorithms for *de novo* assembly of bacterial transcriptomes. The algorithms have been implemented in an open-source software system called Rockhopper 2.

We evaluated Rockhopper 2 using one set of DNA-seq data and 12 sets of RNA-seq data corresponding to a range of microorganisms. We found that Rockhopper 2 produced high quality transcriptome assemblies and outperformed other leading assemblers. Rockhopper 2 has several other advantageous features, including a graphical interface and the ability to run on common laptops rather than necessitating a high-performance computing environment. In addition to *de novo* transcriptome assembly, the Rockhopper 2 system integrates algorithms for normalization of data across experiments, quantification of transcript abundance, and testing for differential gene expression. Thus, Rockhopper 2 reduces the initial stages of analysis of large bacterial RNA-seq data sets to a matter of minutes, enabling investigators to spend more time on downstream interpretation of results and extraction of new biological insights.

## Additional file

Additional file 1:
**For the DNA-seq data set and the 12 RNA-seq data sets used in this study, the table provides details on the performance statistics for each of three assemblers when generating assemblies from each data set.**

## Abbreviations

bp: base pair
PCR: polymerase chain reaction
RAM: random-access memory
RMBT: reads mapping back to transcripts
RPKM: reads per kilobase per million
SRA: Sequence Read Archive
